# Motor Imagery EEG Classification Using Capsule Networks†

**DOI:** 10.3390/s19132854

**Published:** 2019-06-27

**Authors:** Kwon-Woo Ha, Jin-Woo Jeong

**Affiliations:** Department of Computer Engineering, Kumoh National Institute of Technology, Gumi 39177, Korea

**Keywords:** brain-computer interface (BCI), capsule network, deep learning, electroencephalogram (EEG), motor imagery classification

## Abstract

Various convolutional neural network (CNN)-based approaches have been recently proposed to improve the performance of motor imagery based-brain-computer interfaces (BCIs). However, the classification accuracy of CNNs is compromised when target data are distorted. Specifically for motor imagery electroencephalogram (EEG), the measured signals, even from the same person, are not consistent and can be significantly distorted. To overcome these limitations, we propose to apply a capsule network (CapsNet) for learning various properties of EEG signals, thereby achieving better and more robust performance than previous CNN methods. The proposed CapsNet-based framework classifies the two-class motor imagery, namely right-hand and left-hand movements. The motor imagery EEG signals are first transformed into 2D images using the short-time Fourier transform (STFT) algorithm and then used for training and testing the capsule network. The performance of the proposed framework was evaluated on the BCI competition IV 2b dataset. The proposed framework outperformed state-of-the-art CNN-based methods and various conventional machine learning approaches. The experimental results demonstrate the feasibility of the proposed approach for classification of motor imagery EEG signals.

## 1. Introduction

Recently, various studies to facilitate the user’s interaction with devices have been proposed with the development of artificial intelligence technology. These smart interaction technologies try to utilize gesture-based control, eye tracking and gaze estimation, and interpretation of brain signals to improve user experience [1,2,3,4,5,6,7]. In particular, brain-computer interfaces (BCI) have been widely studied recently. The BCI-based approaches take brain waves as input signals and then decode them, to help people with compromised communication skills and/or serious physical disabilities interact with machines more efficiently and comfortably [8], to support computer-aided medical diagnosis for seizure detection and identification of abnormal EEG signals [6,7], and so on.

BCI is one of the most promising technologies that allows users to directly control computers or smart devices without any physical interaction. A BCI-based system generally records the signals generated by the user’s brain and controls a machine by detecting the user’s intent through pre-processing, feature extraction, and classification of brain signals [9]. Among the various methods to capture the brain activities, electroencephalography (EEG) is commonly used to collect and feed input signals to BCI systems owing to its non-invasiveness and low cost. Some popular signal types in the EEG include event-related potential (ERP), steady state evoked potential (SSEP), and brain waves such as sensorimotor rhythm (SMR), alpha (8–15 Hz), beta (16–31 Hz), and gamma waves (>32 Hz). The ERP consists of P300 and error potential [10], while the SSEP is classified into steady-state visual evoked potential (SSVEP) [11] and steady-state sensory evoked potential (SSSEP) [12]. On the other hand, SMR (also known as the “mu rhythm”) is a well-known rhythm that can be observed after attempted or executed motor-related tasks (e.g., right-hand, left-hand, foot, and tongue); therefore, it is most widely and commonly used in motor imagery-based BCI applications [8]. In the SMR, event-related synchronization / desynchronization (ERD/ERS) are observed in the mu band (8–12 Hz) and the beta band (16–31 Hz) [13,14]. The ERD and ERS are phenomena in which signals are attenuated and restored during motor imagery, respectively; therefore, these can be used as useful patterns for motor imagery-based BCI applications.

Motor imagery (MI)-based BCI systems generally perform the following steps: i) recording and pre-processing EEG signals (e.g., SMR), ii) feature extraction from the measured signals, iii) training classification models, and iv) testing using the trained models. In the earlier days of the MI-based BCI study, researchers mainly focused on feature extraction and classification methods to improve the performance of MI-based BCI systems. A common spatial pattern (CSP) algorithm is the most popular method for feature extraction [15,16]. The CSP algorithm designs spatial filters to maximize the variance difference between two different classes, effectively extracting discriminatory features from two-class motor imagery EEG. However, the CSP has a limitation that the frequency bands should be selected manually depending on individual characteristics. To solve this issue, a filter bank common spatial pattern (FBCSP) algorithm has been proposed [17]. The FBCSP algorithm overcame the frequency band problem by using several different frequency bands in parallel and contributed greatly to the performance improvement of motor imagery EEG classification. For classification, classical machine learning methods such as support vector machines (SVMs), linear discriminant analysis (LDA), and naive Bayes (NB) algorithms have been commonly used [18].

Nevertheless, MI-based BCI systems do not perform satisfactorily in terms of accuracy, and there remains room for improvement. To address this problem, deep learning-based approaches have been proposed recently. Various methods using deep learning approaches such as convolutional neural networks (CNNs) [19] have been developed and applied to EEG domain [20,21,22,23]. The study in [24] presented a way to optimize a CNN network for motor imagery EEG. In that work, two CNNs architectures were proposed and evaluated for various network parameters and configurations. Based on their experimental results, the authors argued that CNN-based methods perform better than classical methods [24].

Despite the remarkable success of CNNs with respect to image classification and computer vision, it is known that they have some limitations [25]. First, CNNs do not work well if test data are distorted (e.g., by tilting or rotation) compared with the training data. Second, CNNs can learn limited spatial information by enlarging the field of view with pooling, but do not account for the core spatial relationships between simple and complex objects. These result in the performance degradation of CNNs in terms of classification accuracy. To overcome these limitations, Hinton et al. proposed a new type of deep neural network architecture, called capsule networks (CapsNet) [25]. The CapsNet uses the concept of capsules that can automatically learn various features (e.g., position, rotation, and width in the image domain) of an entity and considers the core spatial relationships between simple and complex objects. Because of these characteristics, a lot of researchers have recently tried to apply the CapsNet architecture to other complex domains as well as image domains. In particular, the EEG signal data in the BCI domain have a low signal-to-noise ratio (SNR) and the signals measured, even from the same person during the same task on the same day, contain a large amount of inconsistent and unstable information. In addition, the ERD/ERS pattern of SMR occurs in different frequency bands for each person [13]. Based on this, we reason that there are many opportunities in the EEG domain that will benefit from the CapsNet. 

In this paper, we propose a method to apply CapsNet for classification of two-class motor imagery EEG signals. We utilize a CapsNet to automatically learn a variety of features from inconsistent motor imagery EEG signals and show that the CapsNet-based architecture can successfully decode the signals. To this end, we use the short-time Fourier transform (STFT) to convert a motor imagery signal to a 2D image. The STFT algorithm transforms one-dimensional motor imagery signals from each EEG electrode into a two-dimensional image in the time-frequency domain. This allows the alpha and beta frequency bands (i.e., the range of the SMR) to be examined over time while preserving the patterns. The converted 2D images are then provided for training and testing of the CapsNet-based architecture. For the analysis and evaluation of the proposed method, the public dataset called BCI competition IV dataset 2b [26] was used. The proposed method was compared with classical machine learning algorithms as well as state-of-the-art CNN-based methods that are widely used for the motor imagery EEG classification. To the best of our knowledge, the proposed work is the first attempt to apply capsule networks to MI-EEG decoding and classification.

The rest of this paper is organized as follows: Section 2 reviews a dataset and previous approaches to classify motor imagery EEG signals and describes the details of the proposed method. In Section 3, the experimental results are discussed. Finally, we provide discussion and conclusions in Section 4 and Section 5, respectively.

## 2. Methods

### 2.1. Dataset and Experimental Environment 

**Figure 1:**
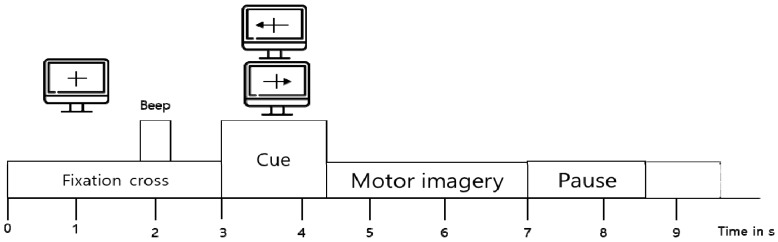
Paradigm of BCI competition IV 2b.

For two-class motor imagery EEG classification, we used the BCI competition IV 2b dataset [26]. The dataset was obtained from nine subjects during a two-class motor imagery task (i.e., left-hand and right-hand) based on the experimental protocol in Figure 1. Three bipolar electrodes (C3, Cz, and C4) were used to record EEG signals, with the sampling frequency of 250 Hz. The EEG signals were band-pass filtered between 0.5 Hz and 100 Hz, and a notch filter was applied at 50 Hz.

The dataset includes five sessions for each subject. The first three sessions consist of training datasets and the remaining sessions consist of test datasets. The first two sessions have each average of 120 trials without visual feedback, and the last three sessions have an average of 160 trials with visual feedback. The entire dataset configuration is described in Table 1.

Our experiments were conducted on a PC workstation equipped with NVidia Pascal Titan X GPU and 1080Ti GPU, 64 GB RAM, and Intel Core i7-6900K (Intel, CA ,USA). Classical machine learning methods were implemented in Python, using the scikit-learn machine learning toolkit [27]. The proposed CapsNet-based method and previous CNN-based methods were implemented using the BrainDecode framework [24] which provides functions related to various EEG preprocessing steps.

### 2.2. Traditional Machine Learning-Based Methods

#### 2.2.1. Filter Bank Common Spatial Pattern

**Figure 2:**
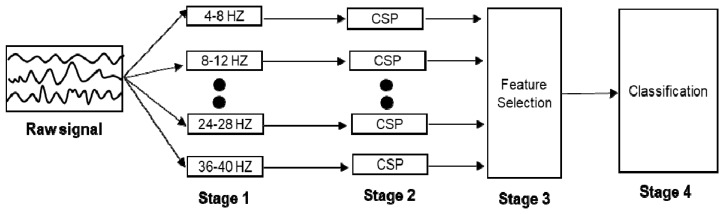
The architecture of FBCSP.

The filter bank common spatial pattern [28] is a widely used for decoding EEG signals as an extension of the CSP algorithm [15,16], and was the best classification method in the BCI competition IV dataset 2b [28]. Therefore, this is an appropriate baseline algorithm for performance evaluation. The CSP algorithm is a spatial filtering algorithm whose objective is to find an important electrode for classification between two different classes. However, the CSP algorithm has several shortcomings. One disadvantage is that the frequency band must be manually selected by the experimenter, according to the individual’s characteristics, so that the classification performance can decrease when the selected frequency is not suitable for the individual. To solve this problem, the FBCSP algorithm, which can automatically detect important frequency bands for each subject, was proposed. The FBCSP consists of four stages, as shown in Figure 2, and as listed below:

(1) In Stage 1, the raw signals are filtered using a filter bank that covers the frequency range of 4–38 Hz in which nine bandpass filters with a bandwidth of 4 Hz each are included (e.g., 4–8 Hz, 8–12 Hz, 12–16 Hz, etc).
(2) In Stage 2, a spatial feature for each frequency band is extracted by the CSP algorithm.
(3) In Stage 3, the best feature among the extracted spatial features is selected based on the mutual information-based best individual feature (MIBIF) extraction algorithm [29].
(4) In Stage 4, various machine learning algorithms, such as SVMs, LDA, and NB algorithm are applied to the extracted features for classification of motor imagery EEG signals.

Nevertheless, there is still room for improvement in terms of efficiency and accuracy, since the FBCSP algorithm relies on the hand-crafted feature engineering such as splitting frequency bands, applying the CSP and the MIBIF method. In addition, more sophisticated methods need to be applied for a classification step to improve the performance.

#### 2.2.2. Classification Methods

Machine learning algorithms commonly used in traditional EEG decoding methods include support vector machine (SVM), linear discriminant analysis (LDA), Naive Bayes (NB), random forest (RF), and K-nearest neighbor (KNN) algorithms. In this section, we briefly introduce the main concept of each algorithm as follows:

(1) A SVM is a supervised learning algorithm based on the concept of a decision boundary; this algorithm can classify linear and non-linear data. The decision boundary is obtained through a margin. The margin is a distance between the decision boundaries that pass through support vectors, and in general the larger the margin, the better the classification.
(2) An LDA is also a supervised classification algorithm based on the concept of the decision boundary. This algorithm looks for the decision boundary that causes the centers (means) of samples from the two different classes to be far from each other and the distributions within the classes to be compact.
(3) A Naïve Bayes classifier is a supervised learning algorithm based on the Bayes theorem under the naive assumption of conditional independence between features. According to the data distribution model, NB algorithms can be categorized into Gaussian NB, Bernoulli NB, and multinomial NB. The Gaussian NB assumes that the continuous data associated with the classes are distributed according to the Gaussian distribution. Similarly, Bernoulli NB and multinomial NB are based on the multivariate Bernoulli distribution and multinomial distribution, respectively. In our experiment, a Gaussian NB classifier was used for comparison.
(4) A decision tree is a supervised learning algorithm that observes a set of training data and then organizes decision rules as a tree structure. Decision trees are simple to understand and to interpret; however, this method is prone to overfitting.
(5) An ensemble is a technique that connects several machine-learning models to create a more powerful model. The ensemble method of decision trees, Random Forest (RF), is an algorithm that obtains a new prediction value through majority voting for the prediction values of the different constituent decision trees.
(6) Finally, the K-nearest neighbor (KNN) is a supervised learning algorithm that classifies data labels using *k* closest examples. The label of a sample is determined by the majority voting over the sample’s neighbors. Distance is typically measured using the Euclidean metric.

### 2.3. Deep Learning-Based Methods

Since classical MI-BCI applications basically depend on feature representations for learning mapping functions from EEG signals to motor commands (e.g., left-hand and right-hand), extraction of useful and meaningful features from the signal is important. In the computer vision field, deep learning approaches such as CNNs have dramatically increased the performance of image understanding and classification [30]. CNNs have proven to be successful because they can automatically detect and learn important features for image understanding and classification [19]. Inspired by this, recent studies have attempted to apply CNNs to EEG understanding and classification tasks. In this Section, we briefly review state-of-the-art CNN-based approaches for motor imagery classification.

#### 2.3.1. Decoding Raw EEG Signals with CNNs

Schirrmeister et al. [24] developed two deep and shallow CNN architectures (hereafter referred to as DeepNet and ShallowNet, respectively) capable of decoding raw EEG signals without hand-crafted features. In addition, they analyzed the performance of CNNs for classification of EEG signals, by varying CNN hyper-parameters such as normalization and activation functions. 

ShallowNet has a shallow network architecture with only two blocks. The first block consists of temporal and spatial convolution layers. Temporal convolution is performed with 40 kernels whose dimension is 1 × 25 and then spatial convolution is conducted with 40 kernels whose dimension is *E* × 1, where *E* is the number of electrodes. In the second block, average pooling is applied and then classification is performed using the Softmax function. The Softmax function takes *K* input values and produces a probability distribution composed of *K* probabilities. The output values of the Softmax function are normalized from 0 to 1, and their sum is always 1. The detailed method for the Softmax function is described in Equation (1):

(1) $$\mathrm{Softmax}\;f{\left(x\right)}_{i}=\frac{\exp(x_{i})}{\sum_{k}\exp(x_{k})}$$

A square and log activation functions are applied to the first block and the second block, respectively. A brief description of the architecture of the shallow CNN model is shown in Table 2. Similarly, the DeepNet contained five blocks. The first block performs temporal and spatial convolution operations and max pooling. The following four blocks consist of a set of convolution and max-pooling layers. In all of the layers except the final dense layer, the rectified linear unit (ReLU) [31] is used as an activation function. For both models, batch normalization and dropout are applied to improve their performance.

In [32], another CNN architecture, called EEGNet, was proposed to handle EEG-based BCI tasks. The EEGNet comprises four building blocks. The first block is learned with sixteen 1D convolutional kernels. In the second and third blocks, four 2D convolutional kernels with zero padding and 2D max-pooling are applied. The fourth layer is a classification layer with the Softmax function. In all of the layers except the final dense layer, the exponential linear unit (ELU) [33] activation function, batch normalization, and dropout are applied to handle overfitting.

#### 2.3.2. Decoding EEG Spectrogram Images with CNNs

A deep learning-based approach such as a CNN generally works well for image understanding and classification; therefore, various attempts have been made to extract 2D image representations from 1D raw signals to solve a time-series classification task with CNNs [34,35]. A common and popular method for this is the short-time Fourier transform (STFT) algorithm, which translates time domain signals into time-frequency domain signals. The detailed method of the STFT algorithm is described in Equation (2):

(2) $$STFT(\tau ,w)=\int x(t)w(t-\tau )e^{-jwt}dt$$

where w(t) is the window function and x(t) is the signal to be transformed. Various windowing functions, such as Hann and Gaussian, can be used as a window function in STFT. As a result of this transformation, a set of 2D spectrogram images can be extracted from the raw signals and used for training and testing CNNs. 

**Figure 3:**
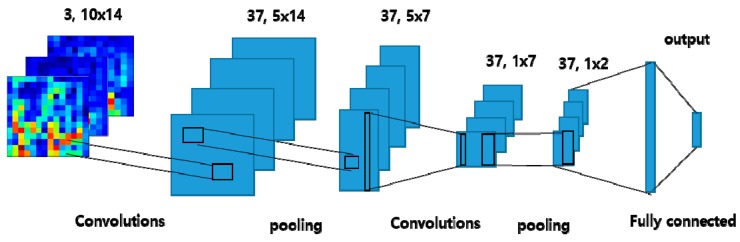
The architecture of CNN-based method.

Lee et al. [34] developed a CNN architecture for decoding two-class motor imagery signals with STFT spectrogram images. First, the STFT is performed with an overlap size of 100 and window size of 128 to obtain 3D data array of size 3 × 65 × 14 from raw EEG signals. Afterwards, the vectors only within the frequency range between mu and beta wave are extracted. That is, the STFT spectrogram images from three electrode channels resulting in 3 × 10 × 14 vectors are used as input. The CNN architecture proposed in [34] is comprised of three layers. The first layer consists of 37, 6 × 1 2D convolutional kernels with 2D max pooling. The second layer is composed of 37, 5 × 1 2D convolutional kernels with 2D max pooling. The final fully connected layer receives the results from the previous layers and derives the probabilities for two output classes using the Softmax function. The ReLU is used as an activation function in the convolution layer and dropout is applied as well. The architecture of the CNN-based method with an STFT spectrogram image is depicted in Figure 3.

Recent CNN-based approaches for MI EEG classification achieved results that were comparable to or better than those obtained using classical CSP-based approaches, even though they do not require extra steps such as feature engineering [24,32,34]. However, as described in the Introduction, CNNs also have limitations in that their performance is compromised on distorted and unstable data. In particular, EEG data are not consistent and stable; therefore, a novel mechanism to handle these characteristics should be studied to improve the overall performance. In this paper, we propose a capsule network-based approach for classification of MI EEG signals and evaluate the feasibility of the proposed framework.

### 2.4. Capsule Network

In this Section, we briefly describe the core concept of capsule networks and the original architecture designed to perform image classification tasks.

**Figure 4:**
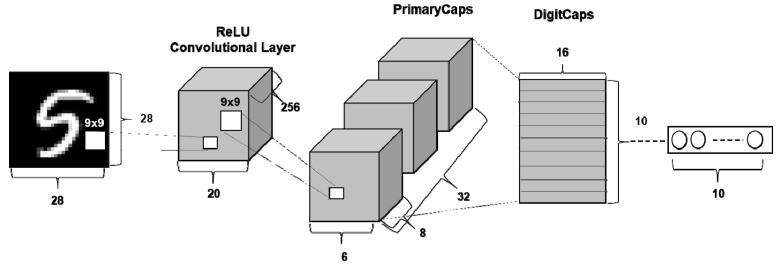
The architecture of original Capsule network [25].

Capsules are groups of neurons for which activity vectors represent various parameters of a specific entity. The length of the activity vectors of a capsule represents the probability that the entity exists, and the orientation of the activity vector represents instantiation parameters. Therefore, a capsule based network architecture is able to represent various properties such as position, size, and rotation using activity vectors. Sabour et al. mentioned that CNNs lose spatial information between objects by using pooling operations, thereby leading to incorrect classification results [25]. Therefore, rather than applying pooling operations, a CapsNet is designed to maintain spatial relationships using a robust and reliable algorithm called “dynamic routing by agreement”. Figure 4 presents the architecture of the original CapsNet for classification of digit characters (i.e., MNIST dataset), which consists of a single convolution layer, followed by a primary capsule layer and a digit capsule layer (output). Dynamic routing is an iterative algorithm that sends the output of a given capsule to appropriate high-level capsules. The iterative algorithm is applied between the primary capsule layer and digit capsule layer. The entire dynamic routing workflow is shown in Table 3. 

For input and output vectors of a capsule, prediction vectors $u^{\wedge }{}_{j|i}$ are calculated by multiplying the output *u_i_* of capsule *i* and weighting metrics *W_ij_*. Capsules *s_j_* are calculated by a weighted sum over all prediction vectors $u^{\wedge }{}_{j|i}$:

(3) $${\hat{u}}_{\;j|i}=W_{\;ij}u_{\;i},s_{\;j}=\sum_{i}\;c_{ij}{\hat{u}}_{\;j|i}$$

where *c_ij_* is the coupling coefficient, calculated by the iterative dynamic routing algorithm process. The coupling coefficient is calculated for capsule *i* and is designed to sum to one using the routing Softmax function:

(4) $$c_{ij=}\frac{\exp(b_{ij})}{\sum_{k}\exp(b_{ik})}$$

where *b_ij_* is a log prior probability that capsule *i* should be coupled to parent capsule *j*, and initial *b_ij_* is initialized to zero. The output *v_j_* is then calculated using a squash function, which acts as a non-linear activation function as follows:

(5) $$v_{j}=\frac{∥s_{j}{∥}^{2}}{1+∥s_{j}{∥}^{2}}\frac{s_{j}}{∥s_{j}∥}$$

The squash function can create vectors close to zero when capsule *s_j_* is small and close to 1 if that capsule is large. The probability of an entity can be expressed as a value between 0 and 1. The log probabilities *b_ij_* are updated in the *r* routing iterations based on agreement. The agreement is calculated as:

(6) $$a_{ij}=v_{j}\cdot {\hat{u}}_{j|i}$$

If the activity vector *v_j_* and prediction vector $u^{\wedge }{}_{j|i}$ are similar, the coupling coefficient increases, but it decreases otherwise. Finally, the loss function of the CapsNet uses the margin loss defined as:

(7) $$L_{k}=T_{k}\max{\left(0,m^{+}-∥v_{k}∥\right)}^{2}+\lambda (1-T_{k})\max{\left(0,∥v_{k}∥-m^{-}\right)}^{2}$$

where *T_k_* is 1 if and only if class *k* is present. The hyper parameters *m*^+^ and *m*^−^ are set to 0.9 and 0.1, respectively. The parameter λ reduces the influence of the loss on those labels that do not belong to the correct class. The value of λ is set to 0.5. 

**Figure 5:**
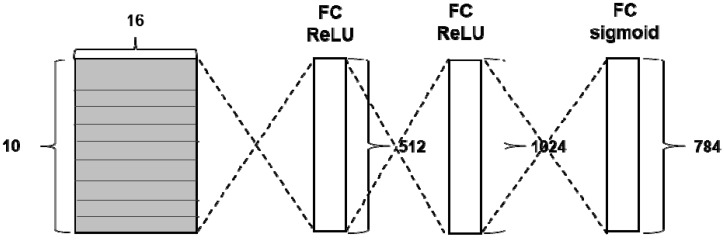
Architecture to reconstruct images from the output capsule layer representation.

As a regularization method of CapsNet, a reconstruction process is performed through three layers fully connected to the output vector, as shown in Figure 5.

The reconstruction loss is obtained using the Euclidean distance between the reconstructed image and the original image. Finally, the total loss is computed by summation of the margin loss and the reconstruction loss as shown in Equation (8). The reconstruction loss is calculated so as not to dominate the margin loss during training; therefore, the weight for the reconstruction loss λ is generally set to 0.0005:

Total loss = margin loss + λ × reconstruction loss(8)

### 2.5. Proposed Method for Decoding EEG with Capsule Networks

**Figure 6:**
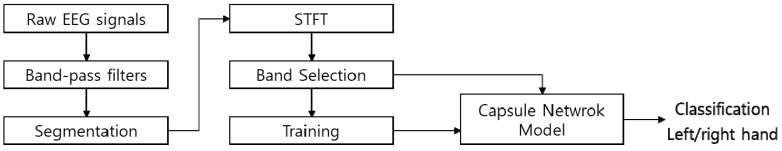
The overall workflow of the proposed system.

The overall workflow of the proposed system is depicted in Figure 6. The EEG signals are divided into alpha (8–15 Hz), beta (16–31 Hz), gamma (> 32 Hz), theta (4–7 Hz), and mu (8–12 Hz) bands, according to their frequency. Depending on the purpose of research, EEG signals in a specific range can be selectively used. When performing a motor imagery task, signal attenuation (ERD) and signal increase phenomenon (ERS) are found in the mu band (8–12 Hz) and beta band (16–31 Hz). Therefore, the combined mu and beta bands (8–31 Hz) are commonly used for motor imagery classification tasks [28]. In our study, raw EEG signals from the dataset were bandpass filtered between 4–38 Hz to cover the mu and beta bands. Afterwards, segmentation was applied to the signals to determine the length of the data to be examined. It is well known that in each MI EEG recording the EEG segment from 0.5 s to 2.5 s after the cue onset produces better classification results [28]. In summary, during this pre-processing step, the EEG signals from the dataset were bandpass filtered between 4–38 Hz and only 2 s of EEG segments (0.5–2.5 s after the cue on-set) were extracted. Since the EEG signals were sampled at 250 Hz, the 2-s-long time step corresponds to 500 samples. 

**Figure 7:**
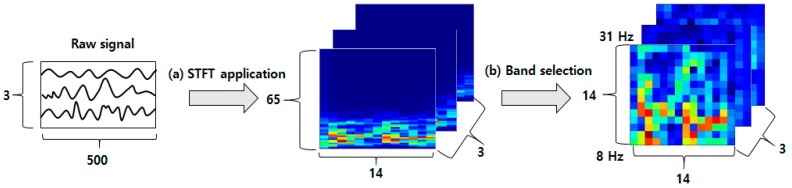
Generation of the 2D images from 1D EEG signals using STFT.

As mentioned above, deep learning-based methods such as CNNs and CapsNets generally work well for image understanding and classification tasks; hence, we apply the STFT algorithm to convert 1D EEG signals to 2D images. The STFT yields a single 2D time-frequency domain spectrogram image for each EEG electrode. For example, if we have EEG signals from *N* electrodes, then *N* 2D spectrogram images can be generated. Figure 7 illustrates the procedure of obtaining spectrogram images using the STFT. After the EEG signals are bandpass filtered and segmented according to the procedure described above, we obtain a data array with the dimensions of *E* × 1 × 500, where *E* is the number of electrodes. The EEG signals in the BCI competition IV 2b dataset were acquired from three electrodes (C3, Cz, and C4); therefore, we have a set of 3 × 1× 500 vectors. The STFT is then performed with an overlap size of 100 and window size of 140 to obtain an array of size 3 × 65 × 14, where the numbers 65 and 14 represent the frequency band and the timestamps, respectively (Figure 7a). After band selection to obtain the segment within beta and mu bands, we obtain 3 channel 14 × 14 2D images (Figure 7b).

**Figure 8:**
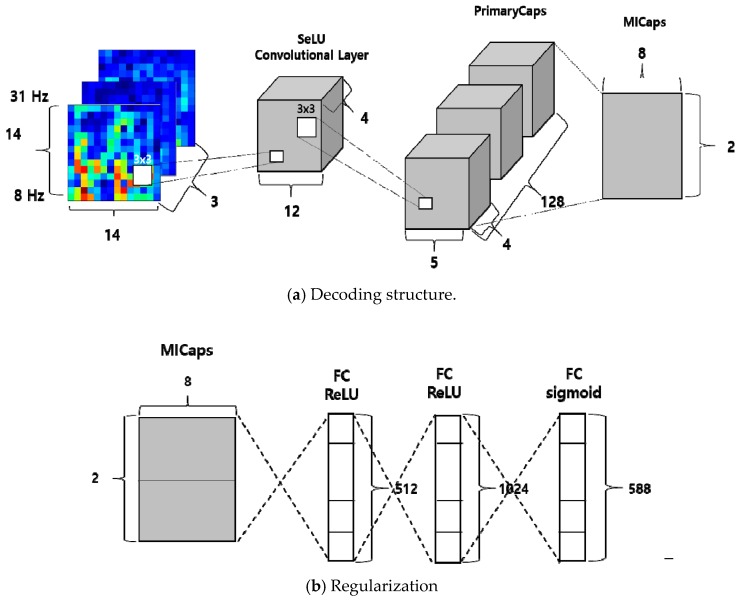
The network architecture of the proposed approach.

Figure 8a shows the architecture of the proposed CapsNet-based approach. Similar to the architecture in the original CapsNet paper [25], our approach also takes 2D images as initial input and generates output vectors through a primary capsule and a motor imagery (MI) capsule. It is worth noting that the parameters of the CapsNet were derived from the optimization tasks that will be discussed in Section 3.1. The summary of the architecture of the proposed CapsNet is as follows:

- A convolutional layer has four kernels with size 3 × 3 with a stride of 1, and yields four feature maps with sizes of 12 × 12. The Selu algorithm [36] is use as the activation function, which is calculated by Equation (9).
- A PrimaryCaps layer consists of 128 channels with four-dimensional capsules (i.e., a primary capsule layer has four convolutional kernels of size 3 × 3 with a stride of 2).
- The last MI-Caps layer has a single 8-dimensional capsule per MI class (i.e., left-hand/ right-hand).
- The decoder consists of three fully connected layers having 512, 1024, and 588 neurons, respectively (Figure 8b). The number of neurons in the last fully connected layer is the same as the number of pixels in the input image (i.e., 3 × 14 × 14 = 588).

(9) $$\mathrm{SELU}\;(x)\;=\;\lambda \left\{ \begin{array}{l} x,x>0\\ a(\exp(x)-1),x<0 \end{array}\right.$$

## 3. Results

### 3.1. Parameter Optimization of the Proposed Network

First, we analyzed various configurations of capsule networks using the BCI competition IV 2b datasets, to determine the optimal network architecture. According to the instruction of the dataset, a classifier was trained and tested for each subject. As explained in Section 2.5, 3 channel STFT spectrogram images (i.e., 3 channel 14 × 14 2D vectors) were used as input for the proposed CapsNet-based method. In addition, the CapsNet-based method was trained in mini-batches of size 50 and using the stochastic gradient descent (SGD) optimizer during 500 epochs. For the SGD optimizer, we set the learning rate to 0.01 and momentum to 0.7. 

The hyper-parameters of the CapsNet to be optimized include: (i) the number of routing iterations (1–3); (ii) application of the reconstruction process (with/without); (iii) the number of convolution channels (256, 128, 64, 32, 16, 8, 4); (iv) the number of the channels in the primary capsule layer (128, 64, 32, 16, 8, 4); (v) the dimension of the primary capsules (32, 16, 8, 4); and vi) the dimension of the MI capsules (16, 8, 4). During the parameter optimization phase, we observed performance changes with the fixed number of routing iterations and reconstruction status while varying other parameters. For example, for the experiment “set 1”, we set the routing number to 3 and enabled reconstruction, and then measured the classification accuracy while varying other parameters such as the capsules dimension and, the number of channels. Overall, we conducted experiments with 6 different scenarios (i.e., routing number: 3/2/1 × reconstruction status: Yes/No).

Table 4 summarizes the classification accuracy of the considered capsule networks with different configurations. In this study, the classification accuracy was calculated as 1 – mis- classification rate (i.e., the number of misclassified trials out of all trials). In Table 4, we only present the network configuration that yielded the best result for each experimental set. For example, we observed the best accuracy of 78.30% when the routing number was 3 and reconstruction was enabled. In this configuration, the number of convolution channels was 4, the number of channels in the primary capsule layer was 128, the dimensionality of the primary capsules was 4, and the dimensionality of the MI capsules was 8.

As can be seen from Table 4, the proposed capsule network-based approach achieves the best average classification accuracy of 78.44% when the number of routing iterations is set to 1, reconstruction is enabled, the number of convolution channels is set to 4, the number of channels in the primary capsule layer is 128, the dimension of a primary capsule is set to 4, and the dimensionality of the MI capsule is 8. Therefore, this configuration was selected as our proposed network architecture as described in Section 2.5. The last row in Table 4 presents the worst classification accuracy and its corresponding network configuration. We found that the CapsNet-based approach achieved the worst accuracy of 69.15% when the number of routing iterations was set to 1, reconstruction was disabled, and the values of all the other parameters were set to 4. Finally, by hyper-parameter optimization, we were able to increase the classification accuracy of the proposed approach by 13.46% (9.29%p), specifically from 69.15% to 78.44%.

**Figure 9:**
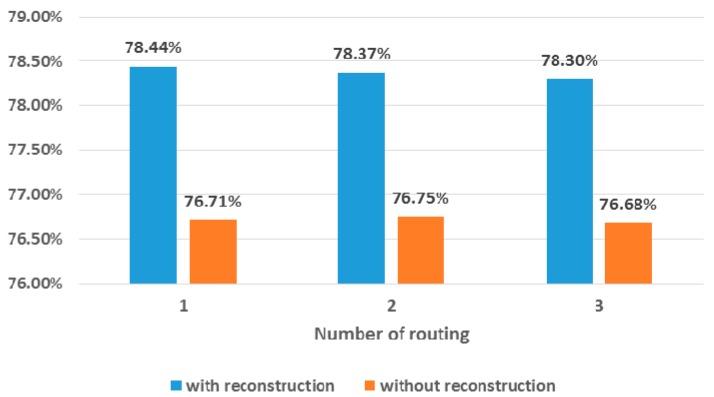
Change of accuracy according to the number of routing iterations and reconstruction step.

We then analyzed the effect of the number of routing iterations and the reconstruction step. To this end, we set the number of convolution channels, the number of channels in the primary capsule layer, the dimensionality of the primary capsule, and the dimensionality of the MI capsule to those from the best configuration and then only changed the number of routing iterations and reconstruction status. Figure 9 summarizes the change in accuracy according to the number of routing iterations and the reconstruction feature. The number of routing iterations hardly affects the network’s classification accuracy. For both cases (i.e., with and without reconstruction), the classification accuracy slightly improved as the number of routing iterations decreased. However, only negligible differences (i.e., 0.07%p for the systems without reconstruction, 0.14%p for the systems with reconstruction) were observed between the classification accuracies. On the other hand, the reconstruction step always contributed to the performance improvement. As Figure 9 shows, performance improved (i.e., maximal 1.73%p and average 1.66%p) regardless of the number of routing iterations.

**Figure 10:**
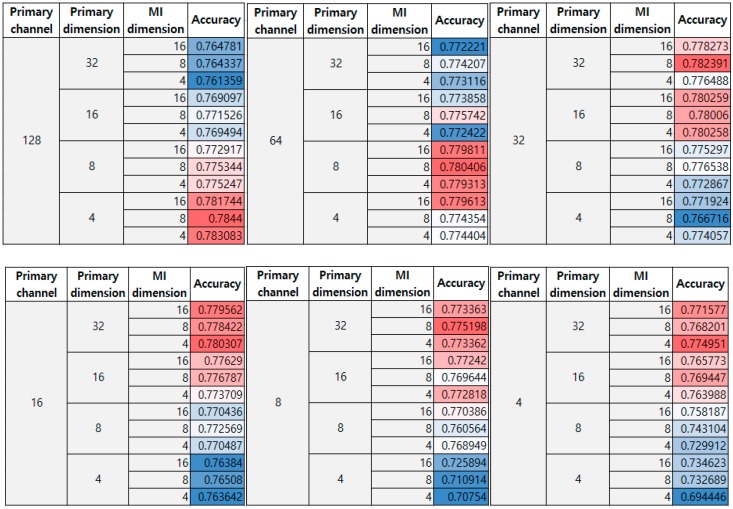
Classification accuracy according to the parameters of the proposed network.

We then investigated the effect of the number of channels in the primary capsule layer, the dimensionality of the primary capsule, and the dimensionality of the MI capsule on the performance of the proposed network. For this analysis, the number of routing iterations was set to 1 and the reconstruction feature was enabled (i.e., best configuration). Figure 10 summarizes the change in the classification accuracy with varying parameters. As shown in Figure 10, if the number of channels in the primary capsule layer is relatively high (i.e., 64 or more), the classification accuracy tends to increase with decreasing dimensionality of the primary capsule layer. On the other hand, if the number of primary channels is low (i.e., 32 or less), the classification accuracy tends to decrease with decreasing dimensionality of the primary capsule layer. Based on these results, we posited that if the number of channels in the primary capsule layer is large, decreasing the dimensionality of each primary capsule improves the network’s performance. However, if the number of channels in the primary capsule layer is small, higher dimensionality for primary capsule is required to capture the characteristics. On the other hand, the dimensionality of the MI capsules does not affect the classification accuracy in most cases except for the case in which the number of channels in the primary capsule layer is 4.

**Figure 11:**
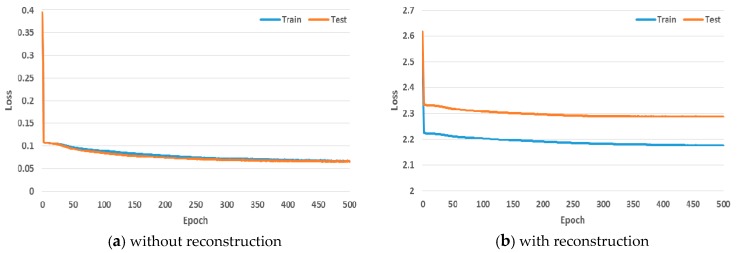
The change of loss during training and testing.

Finally, Figure 11 determines whether the proposed CapsNet-based approach can be well trained in the EEG domain with STFT images. As can be seen from Figure 11a, the loss for the system without reconstruction during training and testing decreases rapidly within the 5 epochs and gradually converges to 0. Similarly, the loss for the system with reconstruction gradually converges to 2.1 (Figure 11b).

### 3.2. Comparison with Baseline Methods

For performance evaluation, we compared the classification accuracy of the proposed CapsNet-based method with those of state-of-the-art CNN-based methods and classical machine learning approaches. As input for training and testing, raw signal-based approaches (e.g., FBCSP, ShallowNet, DeepNet) used 3 × 1 × 500 EEG signal vectors. The STFT-based methods used STFT spectrum images of 3 channel 14 × 14 2D vectors as input.

Table 5 shows the classification accuracy of classical machine learning-based approaches. We show the classification accuracy for each subject, as well as the average classification accuracy across all subjects. As can be seen from Table 5, the SVM outperforms all the other machine learning methods with the average classification accuracy of 72.28%, while the LDA method is the worst with the average classification accuracy of 62.23%. Specifically, the SVM outperforms all the other classifiers except for subjects No.1, No.2, No.3, and No.5. On the other hand, the average classification accuracy of the FBCSP is 67.21%, which is different from the result of the BCI competition. The FBCSP applied to the BCI competition performed best with the handcrafted features (e.g., manually selected training sessions and time periods) for each subject. However, it is impractical to find such an optimal handcrafted feature for each new subject; therefore, all the methods were evaluated under the same conditions in our experiments.

The comparison of the classification accuracies of the proposed CapsNet-based method and other CNN-based methods is presented in Table 6. The SVM was chosen as the representative classical machine learning approach owing to its outstanding performance among the methods. Also, we re-implemented the CNN model [34] in Table 6, which was described in Section 2.3.2 as a CNN baseline model.

As shown in Table 6, the proposed CapsNet-based approach outperforms all the other methods with the average classification accuracy of 78.44%. The proposed approach outperforms the SVM method with the average performance improvement of 6.16%p. In particular, the CapsNet-based method works better than the SVM except for subjects No.2 and No.7. Furthermore, the proposed CapsNet-based method achieved the average performance improvement of 2.92%p over the CNN-based methods. More specifically, the proposed CapsNet-based method demonstrated a considerably better performance (average improvement of 6.66%) for subjects No.1, No.6, No.8, and No.9, compared with the other CNN-based approaches. For subjects No.3, No.4, and No.7, the performance of the proposed was comparable to that of the winner algorithm (i.e., the method of a bolded accuracy value) for each subject. The average performance difference between the proposed method and the winner algorithm was just 0.7%. However, for subjects No.2 and No.5, the EEGNet produced the best result with the accuracy of 58.21% and 86.87%, respectively, which is higher than the proposed method (55.71% and 83.12%).

It is to be noted that the CNN-based approaches (i.e., ShallowNet, DeepNet, EEGNet, and CNN [34]) outperform the proposed work for some subjects, such as subjects No.2, No.3, No.5, and No.7. This implies that the proposed CapsNet-based architecture sometimes fails to capture better features and patterns compared with CNN-based approaches. In the computer vision field, it has been reported that well-designed and tuned CNN architectures still produce slightly better performance than capsule networks for some image classification tasks [37,38]. The experimental results in this study seem to resemble this phenomenon for some cases, which indicates that more investigation of architectural extension to the CapsNet-based approach is required.

**Figure 12:**
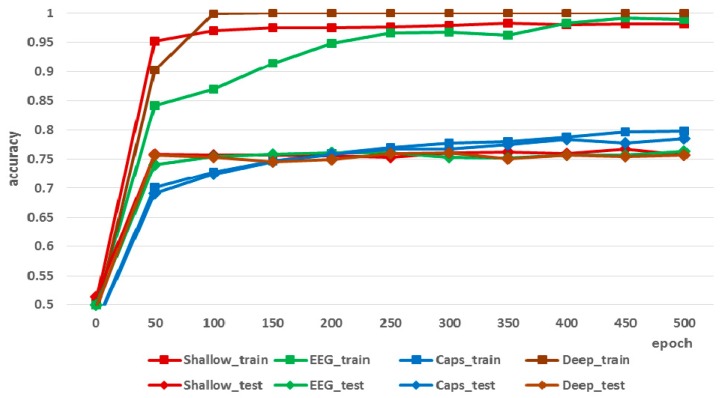
Change in classification accuracy during training and testing according to the epochs.

Afterwards, we analyzed changes in the classification accuracy during training and testing according to the epochs. Figure 12 shows that, the average classification accuracy of the CNN-based methods during training converged to almost 1.00 at 500 epochs, which was substantially better than the result for the proposed CapsNet-based method (0.79). However, it is worth noting that the classification accuracy of the CapsNet-based method during the test was higher than that of the CNN-based methods. This indicates that the CNN-based methods tend to overfit the training dataset. In the BCI domain, avoiding overfitting is one of the most important and challenging issues, because, as mentioned above, even the EEG signals measured from the same person on the same day are inconsistent and unstable.

**Figure 13:**
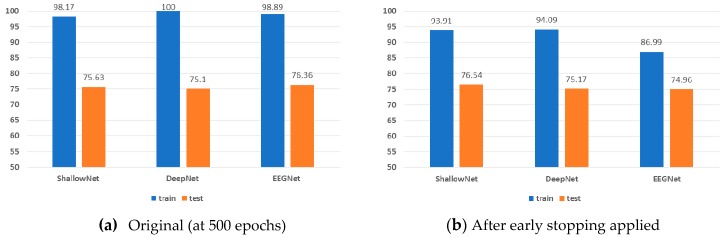
Classification accuracy during training and testing before and after early stopping.

Figure 13 illustrates how the classification accuracy of the CNN-based methods changes if a learning process is terminated earlier. To this end, we applied an early stopping strategy, in which a learning process is terminated if the validation loss of a network does not decrease for a while. After applying the early stopping strategy, the learning processes of ShallowNet, DeepNet, and EEGNet were terminated at 265, 238, and 270 epochs on average, respectively. From Figure 13, we can draw the following conclusions. First, over-learning (i.e., over-fitting resulting from a long learning process) occurs for all CNN-based methods. Compared with the original result (Figure 13a), the gap between the training accuracy and the testing accuracy decreases after early stopping is applied (i.e., from 23.32%p to 16.11%p; refer to Figure 13b). Second, the early stopping strategy affects the performance of each model differently. The testing accuracy of ShallowNet increased from 75.63% to 76.54% while EEGNet produced worse results (i.e., from 76.36% to 74.96%). Conversely, the classification performance of DeepNet was only slightly affected by the early stopping strategy. Third, even when early stopping was applied to the CNN-based methods, the proposed CapsNet-based approach still produced the best performance, without over-fitting.

Finally, we compared the training and testing time for the CNN-based approaches and the proposed CapsNet-based method per subject. As can be seen in Table 7, there are no considerable differences in terms of testing times. All the methods can handle a binary classification task of 320 trials (i.e., 1 epoch) within 0.5 seconds. Conversely, training a network model generally requires more time. However, because training a classification model is usually done offline, all the approaches mentioned in Table 7 can be successfully used online for BCI experiments. Even if the training process should be performed online, most approaches (except DeepNet) require less than 1 min, which is still acceptable in practice.

## 4. Discussion

Through extensive quantitative experiments, we first confirmed the feasibility of the CapsNet-based approach in EEG domain and then validated the efficiency and effectiveness of the proposed method compared to baseline approaches. From the experimental results, we can see that capsule networks successfully learn important features from MI-EEG signals, thereby improving the overall performance. However, there still remain several challenging issues to address. These issues are described below. 

First, the time-frequency domain spectrogram images extracted using the STFT were used as input for training the proposed network. The STFT algorithm is widely used for preprocessing time series signals such as speech and EEG. However, it is well known that the STFT cannot represent various resolutions because of the fixed window length. Therefore, we plan to consider the use of wavelet methods, which can better capture various resolutions, to improve the classification accuracy of the proposed system. Furthermore, we will investigate a method to exploit raw MI-EEG signals without any handcraft feature engineering as well.

Second, we found the optimal network configuration by hyper-parameter tuning. However, there is a limitation in that we only considered the optimization of network parameters, rather than the architecture of capsule networks. In this sense, we believe that the current form of the proposed CapsNet-based approach has limited capability to detect discriminative patterns or features from EEG signals, even though it generally outperforms other baseline methods in terms of average classification accuracy. As shown in Table 6, the CNN-based methods (i.e., ShallowNet, DeepNet, EEGNet, and CNN [34]) sometimes outperform the CapsNet-based approach for subjects No.2, No.3, No.5, and No.7. It is expected that various architectural extensions, such as ensemble, adding more convolution layers, changing the squash function, stacking more primary or intermediate capsule layers, and adopting residual connections [39], can be applied to the CapsNet approach; however, the effects of these extensions for the EEG domain are not clear. Therefore, we will attempt to extend the architecture of capsule networks to determine whether the architectural change can affect the overall performance of the system.

Third, we extracted EEG segments from motor imagery EEG signals using well-known frequency band and time steps. However, fixed-sized segments cannot capture useful features since active frequency bands and time steps for a motor imagery task depend on the analyzed individual. It is also a reasonable assumption that the most discriminative ERS/ERD effects can be differently observed for each subject. This implies that the best combination of electrodes may be different for each subject. To handle these points, we first plan to utilize a feature fusion mechanism to automatically detect informative features for each subject. The feature fusion mechanism is a popular method in the field of image understanding, which allows the integration of features of different scales in a single image [40,41,42]. In particular, this mechanism is generally used to more accurately detect objects on different scales. However, the effect of the feature fusion on detection of active time steps and frequency bands is unclear and requires further investigation. Second, to have an advanced spatial filtering capability (i.e., optimization of electrodes to be analyzed) for the CapsNet architecture, we will develop attention models that can be used for EEG decoding. The attention mechanism has been used to adaptively select important image locations or regions for image processing [41,43], or to selectively weight important words for natural language processing [44]. These mechanisms have proven to be very useful for semantic segmentation, image generation, and machine translation. Our future work on feature fusion and attention mechanisms will reveal how advanced deep learning optimization techniques can affect EEG classification tasks.

As discussed in this Section, the usage of capsule networks in the EEG domain can be improved in several directions. We will address the aforementioned issues to improve the performance of motor imagery EEG classification tasks. 

## 5. Conclusions

In this paper, we proposed a novel approach for classification of two-class motor imagery EEG signals using capsule networks. As input for the proposed method, a set of STFT spectrogram images extracted from raw EEG signals was used. During the CapsNet routing process, the entire network was trained to conduct a classification task. We analyzed and optimized the configuration of the proposed CapsNet architecture with various parameters, such as the number of channels and the number of routing iterations. To evaluate the performance of the proposed approach, we used the BCI competition IV 2b dataset. In the experiment, we first validated the feasibility of the proposed approach and then compared it with other state-of-the-art methods in terms of classification accuracy and efficiency. The experimental results demonstrated that the classification accuracy of the proposed method is better than the classical methods and state-of-the-art CNN-based methods. In addition, we compared the training and testing time for each method and confirmed that the CNN and CapsNet-based methods are available for online use in BCI experiments. 

Despite the successful achievement of the proposed approach, there still remain open issues that need to be addressed in the future. First, as discussed in the Discussion section, our approach can be improved by adopting a variety of novel optimization techniques for hyper-parameter and network structures. We will investigate how recent deep learning techniques can improve the performance of BCI applications. Second, it is also worth investigating if the CapsNet-based method can be applied to more complex tasks (i.e., with more subjects, electrodes, and class labels). Third, we plan to investigate relationships among features, subjects, performance, and layers of the CapsNet architecture. To this end, a method to effectively visualize these relationships will be studied. It is expected that understanding the relationships between the aforementioned components through visualization will enable more meaningful insights. Finally, for more practical BCI applications, various aspects, including accuracy, efficiency, and usability, must be considered together. Our future study will address how to build a compact classification model, as well as how to design a more comfortable and easy-to-use hardware prototype.

## Figures and Tables

**Table 1:** The entire dataset about BCI competition IV 2b.

Subject	1	2	3	4	5	6	7	8	9
Training set	400	400	400	420	420	400	400	440	400
Test set	320	280	320	320	320	320	320	320	320

**Table 2:** The architecture of ShallowNet.

Block	Input	Operation	Output
1	3 × 500	40 × conv2D (1 × 25)	40 × 3 × 476
	40 × 3 × 476	40 × conv2D (*E* × 1)	40 × 1 × 476
	40 × 1 × 476	BatchNorm2D	40 × 1 × 476
	40 × 1 × 476	X^2^-activation	40 × 1 × 476
2	40 × 1 × 476	Reshape	1 × 40 × 476
	1 × 40 × 476	Avepool2D (1 × 75)	1 × 40 × 5
	1 × 40 × 5	Log(x)-activation	1 × 40 × 5
Dense	1 × 40 × 5	Flatten	200
	200	Softmax	2

* *E* is the number of electrodes.

**Table 3:** Dynamic routing algorithm.

**Routing Algorithm**			
1: **Procedure** ROUTING ($u^{\wedge }{}_{j|i}$, *r*, *I*) 2: For all capsule *i* in layer *I* and capsule *j* in layer (*I* +1): *b_ij_* ← 0 3: **FOR** *r* iterations **DO** 4: for all capsule *i* in layer *I*: c_i_ ← sotftmax(b_i_) 5: for all capsule *j* in layer (*I* +1): *s_j_* ←$\sum_{i}c_{ij}u^{\wedge }{}_{j|i}$ 6: for all capsule *j* in layer (*I* +1): $v_{j}$ ← squash($s_{j}$) 7: for all capsule *i* in layer *I* and capsule *j* in layer (*I* +1): $b_{ij}$ ← $b_{ij}$ + $u^{\wedge }{}_{j|i}$.$v_{j}$ 8: Return $v_{j}$			

**Table 4:** Classification accuracy with different network configurations.

Routing	Reconstruction	Conv#	Pri#	Pri_Dim	MI_Dim	Accuracy
3	No	256	128	4	16	77.69%
	Yes	4	128	4	8	78.30%
2	No	256	4	16	4	77.66%
	Yes	4	128	4	8	78.37%
1	No	256	64	4	4	77.55%
	Yes	4	128	4	8	78.44%
1	No	4	4	4	4	69.15%

* The number of convolutional channels (Conv#), the number of primary capsules (Pri#), the dimension of primary and MI capsules (Pri_Dim/MI_Dim).

**Table 5:** Classification accuracy of traditional machine learning methods (%).

Subject	FBCSP	KNN	RF	LDA	SVM	NB
1	**73.5**	71.6	70.8	63.8	70.1	73.1
2	59.4	51.1	56.2	52	56.4	52
3	**61.9**	52.8	55.3	49	53.8	53.6
4	71.5	91	92.1	82.9	**94**	92.3
5	61.4	65.2	80.8	73.6	79	**83.4**
6	70.1	61.3	66.8	61.6	**75.1**	68
7	69.6	71.1	73.6	59.6	**76.1**	74.4
8	62	67.1	71.6	56.8	**71.9**	69.9
9	**75.5**	68.6	73.2	60.8	74.2	71.7
Average	67.21	66.64	71.15	62.23	**72.28**	70.8

**Table 6:** Classification accuracy of CNN based methods and the proposed method (%).

Subject	SVM	ShallowNet	DeepNet	EEGNet	CNN [34]	CapsNet (Proposed)
1	70.1	71.56	67.25	67.18	69.78	**78.75**
2	56.4	53.57	56.10	**58.21**	54.75	55.71
3	53.8	53.12	54.87	**55.62**	52.88	55
4	94	**95.93**	94.52	95.31	95.31	**95.93**
5	79	85	84.59	**86.87**	85.91	83.12
6	75.1	76.87	74.46	77.5	78.03	**83.43**
7	76.1	76.56	**77.03**	76.87	69.75	75.62
8	71.9	85.93	87.75	89.68	87.56	**91.25**
9	74.2	82.18	79.25	80	80.91	**87.18**
Average	72.28	75.63	75.10	76.36	74.99	**78.44**

**Table 7:** Training and testing time for each subject (unit: s).

Parameter	ShallowNet	DeepNet	EEGNet	CNN [34]	CapsNet (Proposed)
Training time/epoch	0.03	0.26	0.05	0.02	0.07
Training time/500 epochs (with early stopping)	12.60 (6.65)	130.68 (62.21)	25.13 (13.57)	8.11	37.47
Testing time/epoch	0.03	0.44	0.02	0.01	0.05
